# Tetranectin as a Potential Biomarker for Stable Coronary Artery Disease

**DOI:** 10.1038/srep17632

**Published:** 2015-12-01

**Authors:** Yanjia Chen, Hui Han, Xiaoxiang Yan, Fenghua Ding, Xiuxiu Su, Haibo Wang, Qiujing Chen, Lin Lu, Ruiyan Zhang, Wei Jin

**Affiliations:** 1 Department of Cardiology, Rui Jin Hospital Shanghai Jiao Tong University School of Medicine, Shanghai, China; 2 Institute of Cardiovascular Diseases Shanghai Jiao Tong University School of Medicine, Shanghai, China

## Abstract

This cross-sectional study tested the hypothesis that decreased serum levels of tetranectin (TN), a regulator of the fibrinolysis and proteolytic system, is associated with the presence and severity of CAD. We conducted a systematic serological and immunohistochemical (IHC) analysis to respectively compare the TN levels in serum and artery samples in CAD patients and healthy controls. Our results showed that serum levels of TN were significantly lower in patients with CAD than in healthy controls. Further analysis *via* trend tests revealed that serum TN levels correlated with the number of diseased arteries. Besides, the multivariate logistic regression model revealed TN as an independent factor associated with the presence of CAD. Additionally, IHC analysis showed that TN expression was significantly higher in atherosclerotic arteries as compared to healthy control tissues. In conclusion, our study suggests that increased serum TN level is associated with the presence and severity of diseased coronary arteries in patients with stable CAD.

Coronary artery disease (CAD), including stable angina, unstable angina, myocardial infarction, and sudden coronary death, is the leading cause of morbidity and mortality globally^1^. Appropriate reperfusion and revascularization strategies, such as thrombolysis therapy, percutaneous coronary intervention, and coronary artery bypass grafting typically improve the quality of life for CAD patients. Clinically, the identification of comprehensive biomarkers has been deemed as a fundamental risk management strategy, since a more accurate assessment of CAD risk will allow for earlier cardio-protective therapeutics, which might potentially delay disease onset and prevent the occurrence of major adverse cardiac events.

Abnormal changes of coagulation and fibrinolysis system play a vital role during the progression of CAD^2^,^3^. Tetranectin (TN), composed of three identical and non-covalently linked 20 kDa subunits, is thought to regulate the fibrinolysis and proteolytic procedures^4^,^5^. During these processes, TN binds specifically to kringle 4 of circulating plasminogen, resulting in an enhanced activation of plasminogen into plasmin. TN has shown to be a potential biomarker for Parkinson’s disease, epilepsy, and prognosis in several types of cancers (such as ovarian, oral, and bladder cancers)^6^,^7^,^8^,^9^,^10^. The precise mechanisms of TN in these diseases are still under investigation. Notably, a recent proteomics study discovered that the serum level of TN was among the predictors of atherosclerotic cardiovascular disease after adjusting for established risk factors^11^.

Although the pathogenic role of TN in the progression of CAD is suggested, there has been no direct clinical evidence focusing on the relationship between circulating TN levels and severity of stable CAD. Thus, in the present study, we examined whether serum TN expression levels correlated with the presence or severity of vascular lesions of stable CAD, as confirmed by elective coronary angiography.

## Methods

The study was conducted in accordance with the principles of the Declaration of Helsinki and approved by the Ethics Review Committee of Ruijin Hospital, Shanghai Jiao Tong University School of Medicine. Written informed consents were obtained from all subjects prior to their inclusion in the study.

### Study Population

**Figure 1:**
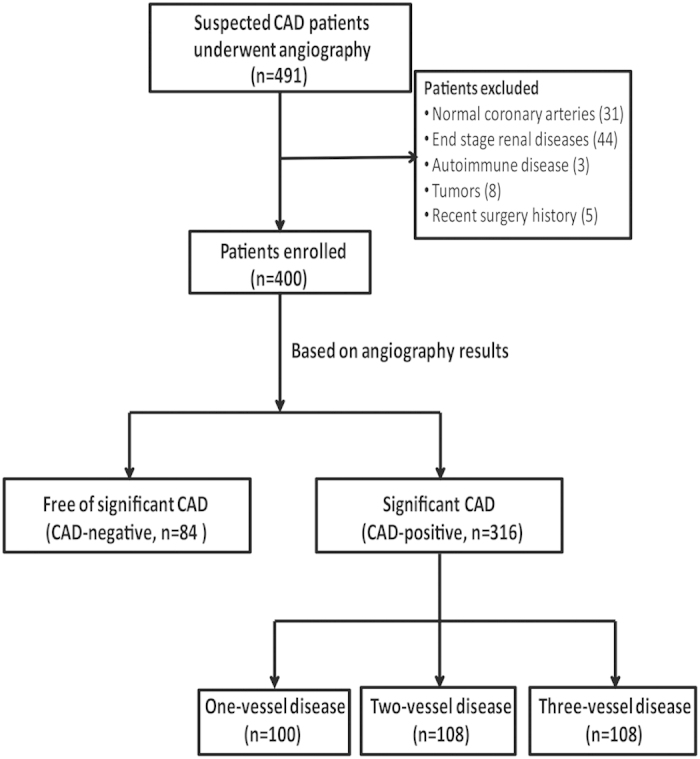
Flow chart of patient enrollment. CAD, coronary artery disease.

A total of 491 patients with suspected CAD undergoing selective coronary angiography between December 2013 and May 2014 were consecutively recruited. Any patient with myocardial infarction within 6 months, those with unstable angina who had angina pain at rest within one month, or those with a history of prior coronary revascularization were excluded from the study. To avoid confounding variables, we excluded individuals with normal coronary arteries (31), end stage renal diseases (44), autoimmune diseases (3), tumors (8), and a recent surgery history (5). Of the eligible 400 subjects who were examined by angiography, 316 patients were diagnosed with significant CAD (CAD-positive) while the rest 84 were not (CAD-negative). According to coronary angiography results, 316 CAD patients were further divided into three groups based on the number of diseased coronary arteries (one-, two-, or three-vessel disease) (Fig. 1). Group I included 100 CAD patients with one-vessel disease (66 men, 34 women, mean age 65 ± 9 years). Group II consisted of 108 patients presenting two-vessel disease (80 men, 28 women, mean age 66 ± 12 years), and Group III comprised of 108 CAD patients with three-vessel disease (92 men, 16 women, mean age 66 ± 9 years). Stable angina was diagnosed according to the criteria recommended by the American College of Cardiology/American Heart Association^12^. At the time of coronary angiography, the information acquired included age, gender, family history, blood pressure, and assessment of risk factors. Cardiac medications taken at study entry, including β-blockers, angiotensin-converting enzyme inhibitors (ACEIs) or angiotensin-receptor blockers (ARBs), aspirin, clopidogrel and nitrates, were recorded. For study purpose, a total of 96 healthy subjects were enrolled as control population.

### Specimen Collection

Human coronary artery specimens were collected from 3 patients with diffuse coronary lesions undergoing endarterectomy. Internal mammary artery (IMA) specimens were obtained from patients who had undergone coronary artery bypass grafting (n = 3). Tissue samples were fixed in 4% paraformaldehyde and embedded in paraffin. Serial sections (6-μm thick) were prepared and stored at room temperature until use.

### Immunohistochemistry (IHC) analysis

For evaluation of TN expression, IHC staining for TN was performed on the artery samples. Serial paraformaldehyde-fixed sections were mounted onto slides, dewaxed, rehydrated, and washed in PBS. Incubation with the anti-rabbit TN antibody (1:500, Abcam, Cambridge, England) was performed in a humidified chamber at 4 °C for 12 hours. After incubation with biotinylated secondary antibody followed by avidin-biotin amplification, the slides were incubated with 3, 3′-diaminobenzidine (DAB) and counterstained with hematoxylin, staining was visualized as a brown precipitate. All images were captured and analyzed by computer-assisted morphometric analysis by using an automated image analysis system (Image-Pro Plus 6.0; Media Cybernetics, Bethesda, Maryland, USA).

### Coronary angiography

Selective coronary angiography was performed through radial or femoral artery approach and the findings were interpreted by at least two experienced cardiologists blinded to the study protocol. Significant CAD was diagnosed visually if luminal diameter narrowing ≥50% was presented at a major epicardial coronary artery^13^. Particularly, left main coronary artery narrowing ≥50% was considered as two-vessel disease.

### Biochemical investigation

For each enrolled subject, blood samples were collected after an overnight fasting. All the samples were analyzed by the laboratory department of our hospital for a biochemical profile. Specifically, serum glucose, platelets, liver function, blood urea nitrogen (BUN), serum creatinine (Scr), uric acid (UA), triglycerides (TG), total cholesterol (TC), high-density lipoprotein cholesterol (HDL-C), low-density lipoprotein-cholesterol (LDL-C) and lipoprotein (a) (Lp(a)) were measured by a Hitachi 912 Analyzer (Roche Diagnostics, Germany). The rest samples were centrifuged at 2000 rpm for 20 min to collect the serum samples, which were then stored at −80 °C until further analysis. Serum TN levels were measured using commercially available human TN ELISA kit (My Biosource, California, USA). No significant cross-reactivity or interference between human TN and analogues was observed. TN measurements were performed by an experienced investigator blinded to subjects’ clinical information and status.

### Statistical analysis

Analysis was carried out with SPSS software (V.13.0, SPSS Inc, Chicago, Illinois, USA). Continuous variables were described as mean ± SEM for normally distributed data or medians with interquartile ranges (IQR) for non-normally distributed data, as appropriate. Categorical data were summarized as frequencies or percentages. Differences in quantitative parameters between groups were assessed by the *t* test (for normally distributed data) or non-parametric test (for non-normally distributed data). Any differences among the three or more groups were evaluated by the analysis of variance with One-Way ANOVA for continuous variables. A logistic regression model using a stepwise backward-selection technique was used to generate a multivariable model to determine the factors associated with the presence of CAD. All analyses used 2-sided tests, a *P* value of <0.05 was considered statistically significant.

## Results

### Baseline Clinical Characteristics

The baseline characteristics of the subjects with CAD and healthy controls are presented in Table 1. Compared with healthy controls, enrolled CAD-positive patients were of older age and had a smaller proportion of females. Additionally, the CAD-positive patients suffered significantly higher levels of liver function, fasting blood glucose, UA, Scr and TG, as well as lower levels of HDL-C (*P* < 0.05). The baseline clinical characteristics of the CAD-negative and CAD-positive groups are summarized in Table 2. Moreover, no significant differences were observed among the CAD-positive subgroups.

### Association between serum levels of TN and CAD

**Figure 2:**
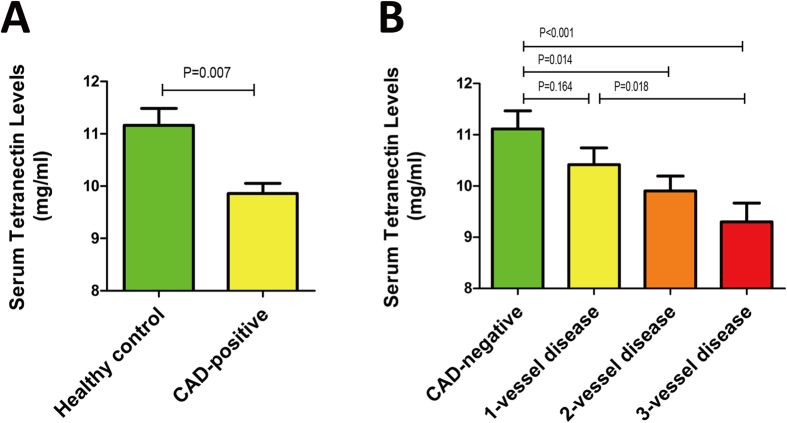
Serum levels of tetranectin (TN) in the study groups. Results are presented as mean±SEM. (**A**) Serum levels of TN in healthy controls and CAD patients. (**B**) Levels of TN in patients with and without significant CAD. A stepwise elevation in the TN levels was found depending on the number of significantly diseased coronary arteries (>50% stenosis). *P* = 0.009 for trend.

Overall, the analysis revealed a significant difference in TN levels between CAD patients and healthy subjects (*P* < 0.05). Specifically, CAD patients had lower levels of TN compared with healthy controls [10.12 ± 3.41 mg/ml *vs*. 11.16 ± 3.17 mg/ml, *P* = 0.007; Fig. 2A]. This finding was consistent with a previous study that examined acute myocardial infarction patients^14^. Notably, a stepwise decrease in the TN levels was found depending on the number of diseased vessels: 11.11 ± 3.27 mg/ml in CAD-negative, 10.41 ± 3.28 mg/ml in one-vessel disease, 9.90 ± 3.03 mg/ml in two-vessel disease, and 9.30 ± 3.84 mg/ml in three-vessel disease. Trend tests revealed that serum TN levels correlated with the number of diseased arteries (*P* = 0.009 for trend, Fig. 2B). Besides, patients with two-, and three-vessel disease had significantly lower TN levels than patients without CAD (*P* = 0.014 and *P* < 0.001, respectively); a significant difference was observed in serum TN levels between one- and three-vessel disease (*P* = 0.018), but not between one- and two-vessel disease (*P* = 0.277).

### Expression of TN in Human Artery Samples

**Figure 3:**
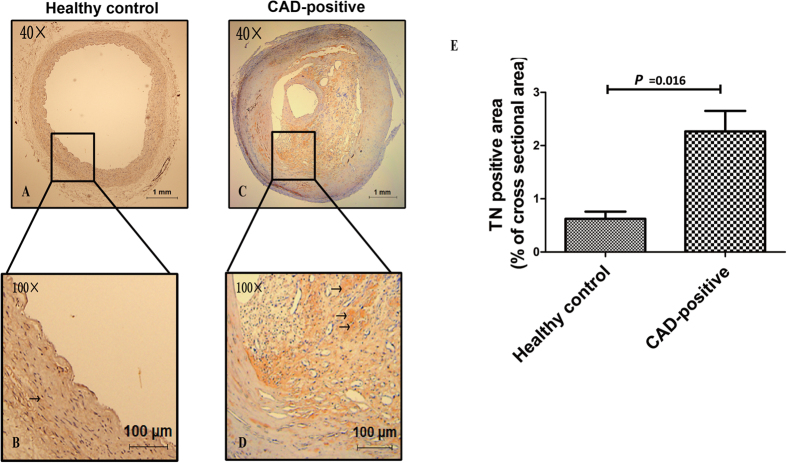
Representative images of immunohistochemical staining for tetranectin (TN) in human normal internal mammary artery and atherosclerotic coronary artery (A and B, normal internal mammary arteries; C and D, atherosclerotic coronary arteries; Magnification: ×40 for A and C; ×100 for B and D; Arrowheads indicate positive staining areas). (**E**) Statistical analysis for TN-positive areas. All of the data were evaluated by measuring the area of positive staining and expressing it as a percentage of the cross sectional area. Data are expressed as mean ± SEM (n = 3).

IHC staining revealed substantial TN expression in the artery specimens in both CAD-positive and healthy control groups (Fig. 3A–D). We further quantified TN expression (by measuring the area of positive staining and expressing it as a percentage of the cross sectional area) using Image Pro Plus 6.0 special image analysis software. The results showed that TN expression was significantly increased in CAD-positive patients, compared with healthy controls [(2.27 ± 0.14) % *vs*. (0.62 ± 0.38) %, *P* = 0.016; Fig. 3E].

### Multivariate analysis

To establish whether TN serum levels were an independent value in predicting the presence of CAD, we performed a logistic regression model using a stepwise backward-selection technique. After adjusting for traditional cardiovascular risk factors (including gender, old age, smoking, hypertension, hyperlipidemia, diabetes mellitus, chronic obstructive pulmonary disease) and all other clinical parameters (including BMI, serum glucose, liver function, BUN, Scr, UA, TG, TC, HDL-C, LDL-C and Lp(a)), the multivariate logistic regression model revealed TN was indeed an independent factor associated with the presence of CAD (Table 3).

## Discussion

Atherosclerosis is the primary reason for stable CAD. Gradual vessel narrowing is a major manifestation in selective coronary angiography^15^. Besides, abnormal coagulation and fibrinolysis is believed to promote localized thrombolytic process in the development of diseased vessels^16^,^17^. Our present study is the first to demonstrate decreased serum TN levels in patients with various degrees of diseased vessels, indicating a linkage of this important regulator of fibrinolysis and proteolysis to the presence and severity of stable CAD.

TN has received considerable attention because of its function in plasminogen activation^18^,^19^. Atherosclerosis is a chronic inflammatory-fibroproliferative arterial disease. During disease development, the imbalance between coagulation and fibrinolytic system plays an essential role^20^,^21^. Consistent with those results, serum TN level was lower in CAD patients than healthy subjects from our investigation. Of note, serum TN levels were independently associated with CAD, from the results of multivariable logistic regression analysis in the model including age, gender, BMI, smoking, hypertension, hyperlipidemia, diabetes mellitus, chronic obstructive pulmonary disease, serum glucose, liver function, BUN, Scr, UA, TG, TC, HDL-C, LDL-C and Lp(a) levels. According to TN’s structural and binding properties, we suspected that the underlying mechanism might be attributed to a high uptake by the formation of atherosclerotic plaques^22^,^23^, which are characterized by lipid, albumin, Lp(a) and fibrin/fibrinogen deposition^11^,^24^,^25^. Consistently, IHC results revealed high expression of TN in human atherosclerotic lesions.

Subgroup analysis further strengthened our assumption. The more severe the coronary arteries presented, the greater change of the serum TN levels was observed. Compared with patients with one-vessel disease, patients suffering three-vessel disease presented a significantly decreased level of serum TN. Three-vessel disease is associated with a heavy load of intracoronary-thrombus^26^, which tends to reflect the degree of inflammatory-fibroproliferative process in atherosclerosis. Thus, atherosclerosis-related endothelial damage might lead to intimal accumulation of TN in complexes with Lp(a) and/or fibrin, thus diminishing serum TN levels^27^.

In conclusion, we observed decreased levels of serum TN in patients suffering stable CAD and an increased level of TN expression in coronary atherosclerotic lesions. It is possible that reduced serum TN levels in CAD patients observed in this study were a result of an increased plaque uptake for this purpose. Despite the clinical association confirmed in this study, additional investigations are necessary for the elucidation of the underlying mechanism of TN on the pathogenesis of atherosclerosis.

### Study Limitations

There are several limitations in the present study. First, the study is cross-sectional for the investigation of the presence and severity of stable CAD, thereby allowing us to detect association, but not to predict outcome. Second, TN serum expression reflects whole body levels, not coronary vessels solely. Third, selective coronary angiography was not performed on the healthy controls enrolled in the study. Therefore, a more comprehensive analysis was not allowed between patients with and without CAD.

## Conclusions

The present study has demonstrated that increased TN levels in serum are associated with the presence of CAD and number of diseased vessels in patients with stable CAD.

## Additional Information

**How to cite this article**: Chen, Y. *et al.* Tetranectin as a Potential Biomarker for Stable Coronary Artery Disease. *Sci. Rep.* **5**, 17632; doi: 10.1038/srep17632 (2015).

## Figures and Tables

**Table 1:** Baseline characteristics of healthy controls and CAD patients.

Variables	Healthy controls (n=96)	CAD patients (n=316)	*P* value
Age (years)	37 ± 10	66 ± 10	<0.001
Male (%)	53 (44.40)	238 (75.31)	<0.001
Glucose (mmol/L)	5.34 ± 0.39	5.61 ± 1.52	0.002
Platelets (10^3^/mm^3^)	232 ± 63	193 ± 59	<0.001
ALT (IU/L)	23.34 (21.13–25.08)	26.19 (24.12–28.65)	0.050
AST (IU/L)	20.25 (19.68–21.47)	27.66 (24.90–31.66)	<0.001
γ-GT (IU/L)	16.22 (14.37–18.56)	27.52 (25.20–30.24)	<0.001
BUN (mmol/L)	4.33 ± 0.82	5.58 ± 2.20	<0.001
SCr (μmol/L)	77.16 ± 13.20	84.50 ± 25.77	<0.001
Uric acid (μmol/L)	274.20 ± 60.33	350.74 ± 106.10	<0.001
TG (mmol/L)	1.10 ± 0.55	1.70 ± 1.06	<0.001
TC (mmol/L)	4.74 ± 0.61	4.07 ± 1.13	<0.001
HDL-C (mmol/L)	1.43 ± 0.31	1.02 ± 0.30	<0.001
LDL-C (mmol/L)	2.65 ± 0.49	2.50±0.95	0.084
Lp(a) (mmol/L)	0.18 (0.14–0.22)	0.26 (0.22–0.31)	0.006

Data are expressed as mean ± SD, median (interquartile range), or frequency counts (percentages), as appropriate. CAD, coronary artery disease; ALT, alanine aminotransferase; AST, aspartate transaminase; γ-GT, gamma-glutamyl transpeptidase; BUN, blood urea nitrogen; SCr, serum creatinine; TG, triglycerides; TC, total cholesterol; HDL-C, high-density lipoprotein-cholesterol; LDL-C, low-density lipoprotein-cholesterol; Lp(a), lipoprotein(a).

**Table 2:** Baseline clinical characteristics in CAD subgroups and patients without significant CAD.

Variables	CAD-negative (n = 84)	1-vessel disease (n = 100)	2-vessel disease (n = 108)	3-vessel disease (n = 108)
Age (years)	62 ± 10	65 ± 9	66 ± 12*	66 ± 9*
Male (%)	49 (58.30)	66 (66.00)*	80 (74.00)*	92 (85.00)*
Etiology				
Hypertension (%)	44 (52.40)	72 (72.00)*	71 (65.74)*	76 (70.37)*
Diabetes mellitus (%)	25 (29.8)	27 (27.00)	31 (28.70)*	35 (32.40)*
Hyperlipidaemia (%)	6 (7.14)	7 (7.00)	10 (9.26)*	6 (5.56)
Systolic BP (mmHg)	132 ± 20	140 ± 18*	134 ± 21	140 ± 21*
Diastolic BP (mmHg)	76 ± 13	78 ± 11	74 ± 11	76 ± 12
Family history (%)	6 (7.00)	10 (10.00)*	19 (17.57)*	11 (10.19)*
Smoking status (%)	14 (16.70)	36 (36.00)*	31 (28.70)*	36 (33.33)*
Leukocytes (10^3^/mL)	6.23 ± 1.27	6.51 ± 1.37	6.52 ± 1.54	7.01 ± 2.20*
Hemoglobin (g/dL)	135.37 ± 13.72	133.90 ± 14.58	131.56 ± 14.41	129 ± 17.00*
Platelets (10^3^/mL)	196.55 ± 63.73	188.55 ± 55.28	194.32 ± 57.37	196 ± 64.00
Glucose (mmol/L)	5.16 ± 1.79	5.44 ± 1.26	5.68 ± 1.65*	5.75 ± 1.49*
HbA1c (%)	5.80 ± 1.00	6.30 ± 1.23*	6.48 ± 1.27*	6.60 ± 1.30*
BUN (mmol/L)	5.45 ± 2.00	5.36 ± 1.70	5.19 ± 1.84	6.21 ± 2.67*
SCr (μmol/L)	78.35 ± 19.96	78.85 ± 20.53	82.65 ± 28.82	91.57 ± 25.52*
Uric acid (μmol/L)	329.55 ± 104.51	352.44 ± 87.27	345.34 ± 117.08	354.57 ± 110.97
TG (mmol/L)	1.67 ± 1.04	1.79 ± 1.21	1.63 ± 1.12	1.67 ± 0.83
TC (mmol/L)	4.11 ± 1.08	4.02 ± 0.94	4.01 ± 1.18	4.17 ± 1.25
HDL-C (mmol/L)	1.25 ± 0.71	1.08 ± 0.28	1.02 ± 0.37*	0.95 ± 0.22*
LDL-C (mmol/L)	2.46 ± 0.82	2.41 ± 0.79	2.44 ± 0.97	2.63 ± 1.04*
Lp(a) (mmol/L)	0.32 (0.18–0.46)	0.18 (0.14–0.21)*	0.27 (0.21–0.34)*	0.33 (0.21–0.44)
Creatine kinase (U/L)	83 (72–94)	108 (94–123)*	124 (91–156)*	94 (81–107)*
D-dimer (mg/L)	0.31 (0–0.39)	0.52 (0–0.87)*	0.64 (0–1.29)*	0.37 (0.27–0.46)
LVEF (%)	64.20 ± 9.09	64.07 ± 7.39	62.64 ± 8.99	61.41 ± 9.14*
Medications on enrollment				
β-blockers (%)	56 (66.67)	75 (75.00)*	92 (85.19)*	95 (87.96)*
ACEI or ARB (%)	36 (42.86)	60 (60.00)*	63 (58.33)*	69 (63.89)*
Clopidogrel (%)	16 (19.05)	68 (68.00)*	92 (93.9)*	78 (79.6)*
Aspirin (%)	56 (66.67)	69 (69.00)*	97 (89.81)*	99 (91.67)*
Statins (%)	50 (59.52)	88 (88.00)*	96 (88.89)*	86 (79.63)*
Nitrates (%)	18 (21.43)	42 (42.00)*	44 (40.74)*	64 (59.26)*

Data are expressed as mean ± SD, median (interquartile range), or frequency counts (percentages), as appropriate. CAD, coronary artery disease; BP, blood pressure; HbA1c, glycated hemoglobin; BUN, blood urea nitrogen; SCr, serum creatinine; TG, triglycerides; TC, total cholesterol; HDL-C, high-density lipoprotein-cholesterol; LDL-C, low-density lipoprotein-cholesterol; Lp(a), lipoprotein(a); LVEF, left ventricular ejection fraction; ACEI, angiotensin-converting enzyme inhibitor; ARB, angiotensin-receptor blocker. *P < 0.05 compared with the CAD-negative group.

**Table 3:** Multivariable stepwise logistic regression analysis for the presence of CAD.

Variable	OR (95% CI)	*P* value
Tetranectin	0.680 (0.491,0.940)	0.020*
Age	1.402 (1.184,1.660)	<0.001*
Male	0.159 (0.016,1.546)	0.113
BMI	0.775 (0.541,1.109)	0.164
Platelets	0.983 (0.963, 1.003)	0.096
Glucose	1.258 (0.472, 3.355)	0.647
ALT	1.034 (0.974, 1.098)	0.269
AST	1.018 (0.929, 1.116)	0.701
γ-GT	1.011 (0.988, 1.035)	0.357
BUN	1.882 (0.668, 5.304)	0.231
SCr	0.951 (0.916, 0.987)	0.008*
Uric acid	1.008 (0.994, 1.022)	0.265
TG	76.799 (3.368,1751.320)	0.007*
TC	0.000 (0.000,0.020)	<0.001*
HDL-C	616.687 (2.917,1.3*10^5^)	0.019*
LDL-C	3.2*10^5^ (96.223,1.1*10^7^)	<0.001*
Lp(a)	5.099 (0.019,1336.635)	0.566
Smoking	0.174 (0.011,2.657)	0.209
Hypertension	0.290 (0.039,2.140)	0.225
Diabetes mellitus	1.909 (0.232,15.691)	0.547
Hyperlipidaemia	0.455 (0.048,4.304)	0.492
COPD	0 (0.000,0.020)	0.999

CAD, coronary artery disease; BMI, Body Mass Index; ALT, alanine aminotransferase; AST, aspartate transaminase; γ-GT, gamma-glutamyl transpeptidase; BUN, blood urea nitrogen; SCr, serum creatinine; TG, triglycerides; TC, total cholesterol; HDL-C, high-density lipoprotein-cholesterol; LDL-C, low-density lipoprotein-cholesterol; Lp(a), lipoprotein(a); COPD, chronic obstructive pulmonary disease.CI, confidence interval; OR, odds ratio; *P < 0.05.
